# Flowers

**Published:** 1861-05

**Authors:** 


### FLOWERS.

THE very sight of what is beautiful tends to purify the heart and elevate the character; while the cultivation of flowers directly promotes physical well-being. The following list of flowering plants was made out by the *Germantown Telegraph* to afford a succession of bloom throughout the season, and with the page about clocks made from flowers, will be regarded with interest by every intelligent reader in the beautiful May. In this connection may be premised a striking exemplification of the instinct of plants by the naturalist Hoare, who placed a bone in the strong, dry clay of a vine border. The vine sent out a leading or tap-root, directly through the clay; the main root threw out fibers, but when it reached the bone it entirely covered it by degrees with the most delicate and minute fibers like lace, each one sucking at a pore in the bone, like a litter of pigs at their dam, as she lies down on the sunny-side of the farm-yard. On this luscious morsel of a marrow-bone would the vine continue to feed as long as any nutriment remained to be extracted. What wonderful analogies there are running through the various forms of animal and vegetable creation, to stimulate curiosity, to gratify research, and, finally, to lead our contemplations from nature, in a feeling of reverence "up to nature's God."

As to the vine spoken of by Hoare, it is worthy of remark that the root went no further than the bone, which it seemed to have literally smelt out, as would a hungry dog, in passing.

### FLOWERING SHRUBS.

Pink Mezereon.
Dwarf double-flowering Almond.
Double Purple Tree Peony.
Chinese White Magnolia. (*Conspicua.*)
Soulange's Magnolia.
Sweet-scented Magnolia. (*M. glauca.*)
White Fringe Tree.
Garland Deutzia. (*D. Scabra.*)
Broad-leaved Laburnum.
Rose Acacia.
Tartarian Tree-Honeysuckle, red and white.
Double White Hawthorn.
Double Pink Hawthorn.
Fragrant Clethra.
Oak-leaved Hydrangea.
Venitian Sumac or Purple Fringe.
Buffalo Berry, (male and female.)
Siberian Lilac.
The Althea or Hibiscus Syriacus.
Colutea Arborescens.
Chinese double-flowering Apple.
Deutzia Gracillis.
All the Spireas.
Snowball, (common though beautiful.)
Dwarf Dogwood.
Pyrus Japonica.
Euonymus, (burning bush.)
Forsythia.
Philadelphus, (Mock Orange.)
Symphora.
Wiegeila Rosea.

### PERRENIAL PLANTS.

Dicentra Spectabilis.
Plumbago.
White and Pink Phlox.
[There are from twenty to thirty common Phloxes, many of them dwarf, of beautiful colors and much admired.]
Companulas.
Chrysanthemums, (summer and fall.)
Double Hollyhocks.
Pæonias, (white and red.)
Iris, (pale blue, very fragrant.)
Sweet William.
Valeriana.
Persian Lilac.

### CLIMBING SHRUBS AND VINES

Some of the finest and hardiest climbing shrubs are the following:

Large flowering Trumpet Creeper.
Queen of the Prairie Rose.
Chinese Glacine, (Wistaria.)
Double Purple Clematis.
Clematis Flamula, Florida and Siboldii.
Monthly Fragrant Honeysuckle.
Chinese Twining Honeysuckle.
Yellow Trumpet Honeysuckle.
Scarlet Trumpet Honeysuckle.
Japan Evergreen Honeysuckle.
Chinese Bignonia.
Virginia Creeper.
Periwinkle, (as a creeper for shady places.)

### CLIMBING ROSES.

Queen of the Prairies.
White Multiflora.
Laura Davoust, (half-hardy.)
Baltimore Belle.

### TRAILING ROSES.

Fellenberg.
Glory of Rosamond.
Monstrosa.
Baron Prevost.
Noisette Superba.
La Reine.

### MONTHLY ROSES.

Hermosa, pink.
Cels, blush and pink.
Devoniensis, creamy white.
Archduchess, pure white.
Giant of Battles, crimson.
Louis Philippe, red.
Souvenir, blush.
Luxemborg, buff.
Queen of Lombardy, deep rose.
Saffrana, yellow buff.
Daily, light Pink.
Prince Albert.
Garibaldi.
Triomphe l'Exposition.
Monthly Cabbage.

